# Fish-seastar facilitation leads to algal forest restoration on protected rocky reefs

**DOI:** 10.1038/srep12409

**Published:** 2015-07-22

**Authors:** Nicola M. Galasso, Chiara Bonaviri, Francesco Di Trapani, Mariagrazia Picciotto, Paola Gianguzza, Davide Agnetta, Fabio Badalamenti

**Affiliations:** 1 CNR-IAMC, via Giovanni da Verrazzano 17, 91014 Castellammare del Golfo, Italy; 2 Dipartimento di Scienze della Terra e del Mare (DiSTeM), Università di Palermo, Via Archirafi 22, I-90123 Palermo, Italy

## Abstract

Although protected areas can lead to recovery of overharvested species, it is much less clear whether the return of certain predator species or a diversity of predator species can lead to re-establishment of important top-down forces that regulate whole ecosystems. Here we report that the algal recovery in a Mediterranean Marine Protected Area did not derive from the increase in the traditional strong predators, but rather from the establishment of a previously unknown interaction between the thermophilic fish *Thalassoma pavo* and the seastar *Marthasterias glacialis*. The interaction resulted in elevated predation rates on sea urchins responsible for algal overgrazing. Manipulative experiments and field observations revealed that the proximity of the seastars triggered an escape response in sea urchins, extending their tube feet. Fishes exploited this behavior by feeding on the exposed tube feet, thus impairing urchin movement, and making them vulnerable to predation by the seastars. These findings suggest that predator diversity generated by MPA establishment can activate positive interactions among predators, with subsequent restoration of the ecosystem structure and function through cascading consumer impacts.

Marine communities are rapidly changing due to anthropogenic impacts. In this context, depletion of predators due to overfishing may lead to degradation of ecosystems through trophic cascades^1^,^2^,^3^,^4^. Recent global climate change is causing shifts in the biogeographical ranges of some species^5^, providing novel assortments of species with potentially new and unexpected effects on ecosystems^6^. New interactions among species and the strengthening of existing ones may lead to unexpected consequences for ecosystem function^7^,^8^.

Banning human activities in Marine Protected Areas (MPAs) usually promotes recovery of targeted predators, especially strong predators such as large fishes and lobsters^9^,^10^,^11^ which, indirectly, leads to ecosystem restoration through trophic cascades. Such effects are by no means certain to occur in all locations however^12^,^13^,^14^,^15^, and responses to protection from fishing may be variable in magnitude, direction and degree of stability^16^. Sometimes no recovery is seen in higher predators on timescales of years, suggesting that ecosystem functioning within MPAs can follow different paths^11^,^14^,^17^,^18^. The establishment of direct and indirect interactions among different predator species may provide an alternative mechanism for controlling prey populations^19^. The potential for new interactions to occur is likely promoted by the increase in diversity across taxa and trophic groups after protection enforcement^10^,^20^. In particular, facilitative interactions among different predators can enhance the predation rate on common species^21^,^22^,^23^,^24^,^25^,^26^,^27^,^28^. Through synergistic facilitation^19^,^29^, weak predators can then control prey populations and restore the ecosystem even in the absence of strong predators. This process may be favored by the arrival of new species, creating novel species assemblages, due to range shifts prompted by climate change^30^.

The control of sea urchin populations by key predators, prompting a regime shift from grazed “barrens” to kelp forest, has been particularly well studied in temperate rocky reef MPAs^31^,^32^,^33^,^34^,^35^,^36^. In the Mediterranean Sea, recovery of sea bream populations (genus *Diplodus*) promotes algal forest growth by predation on the sea urchins *Paracentrotus lividus* and *Arbacia lixula*^17^,^36^. However, in some cases spatial protection alone is not sufficient to restore *Diplodus* populations^37^,^38^. This is the case in the “Ustica Island” MPA, where protection was enforced in 1991. Unlike other Mediterranean MPAs^17^,^36^, at Ustica the upper infralittoral zone developed as a barren following protection enforcement^39^. Until 2007, the sea urchins *P. lividus* and *A. lixula* and encrusting corallines such as *Lithophyllum spp*., *Pseudolithophyllum expansum*, *Lithothamnium spp.* and *Mesophyllum coralloides* dominated the shallow rocky bottom assemblages^40^. In recent years, sea urchins abundance decreased, the forest restored^41^, yet the population of *Diplodus* remained unexpectedly low^14^, following a steady long-term decline^38^. Sea urchin depletion was instead accompanied by an exceptional increase in the density of the seastar *Marthasterias glacialis* in the sea urchin-barren areas^14^ and by an emergent, facultative synergistic interaction between the seastar and the thermophilic fish *Thalassoma pavo,* herein described.

The wrasse *T. pavo* is known to consume small molluscs, crustaceans and juvenile sea urchins^42^. Although this small fish is unable to overcome the defences of adult sea urchins^43^, the congeneric *T. noronhanum* is reported to occasionally tear off parts of the sea urchin tube feet^44^. At the “Ustica Island” MPA, *T. pavo* is abundant on shallow reefs^45^, since it is favoured by the warm water and the steep shores of the island^46^,^47^,^48^,^49^, and possibly also by protection. The seastar *M. glacialis* is usually rare on Mediterranean shallow reefs, being more common in deeper reefs, and has so far been overlooked as a predator of sea urchins due to its preference for bivalves^42^. Sea urchins are able to perceive seastars from a distance of up to 50 cm and successfully flee, hence reducing the success of attacks^50^,^51^. The predatory strength of *M. glacialis* on sea urchins is thus usually weak, but in Ustica MPA, *M. glacialis* is particularly abundant in the barren areas and is an important predator of sea urchins^14^,^52^.

Field observations showed that, in the proximity of *M. glacialis*, sea urchins escape their potential predator by extending their tube feet, lowering their spines and fleeing away from the seastar. At this point, several individuals of *T. pavo* usually gather and repeatedly bit off urchin tube feet. Mutilated sea urchins are then impaired in their movement capacity, and become vulnerable to seastar predation.

We hypothesize that, after MPA implementation, an emergent, synergistic facilitative interaction between the seastar and the fish makes sea urchins vulnerable to seastar predation, thus contributing to population control of sea urchins at “Ustica Island” MPA allowing for consequent recovery of algal forests.

## Results

**Figure 1:**
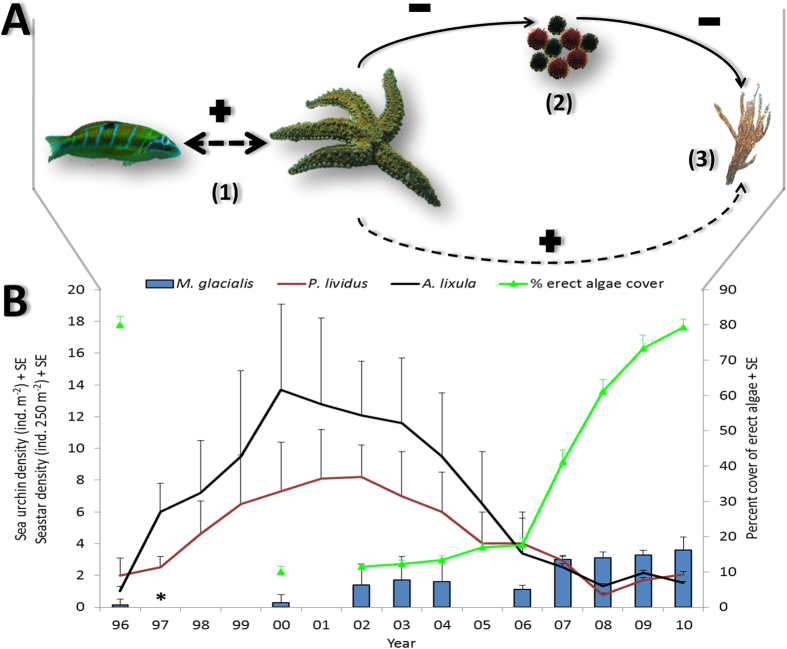
Conceptual model of trophic interactions and their effect on habitat state in Mediterranean rocky reefs. (**A**) Interactions among the seastar *M. glacialis,* the ornate wrasse *T. pavo*, the sea urchins *P. lividus* and *A. lixula* and the algae *Cystoseira spp*. Dotted arrows indicate indirect interactions, whereas solid arrows indicate direct interactions. +/− symbols indicate positive or negative effect of the interaction on the species pointed by the arrow. In Ustica Island the interaction between fish and seastar (1) facilitates predation on sea urchins (2) by the seastar. Urchins are in turn strong grazers and are able to alter algal coverage (3). (**B**) Temporal trends in *M*. *glacialis* densities, sea urchin densities and habitat state in Ustica Island MPA. Red line: *P. lividus*. Black line: *A. lixula.* Blue bars: *M. glacialis.* Green line: algal coverage. Images in figure by F. Di Trapani and P. Gianguzza.

Long-term data showed a clear trend with densities of both *P. lividus* and *A. lixula* enormously increasing after protection enforcement (Fig. 1). This trend reversed from 2000 to 2010 (Fig. 1). The decrease of sea urchins was accompanied by an increase in the population of *M. glacialis* and an extension of the algal coverage. The ecosystem state eventually shifted from barren to *Cystoseira spp*. forest (Fig. 1).

The success of the fish-seastar interaction was in part reflected by the dominance of sea urchins in the diet of large seastars (>27 cm total length). *P. lividus* accounted for 51.3% of captured prey observed and *A. lixula* for 28.2% (Supplementary Table 1). Similarly, tube feet of sea urchins were frequently found in large quantities (mean ± SE = 16.5 ± 4.8) in the stomachs of *T. pavo* (33% of fishes), indicating that the interaction is common throughout the MPA. Through direct observation and video recording, we established that the wrasse removed 39–88% of tube feet from *P. lividus* and 58–84% of tube feet from *A. lixula* during seastar attacks (Supplementary Fig. 1).

**Figure 2:**
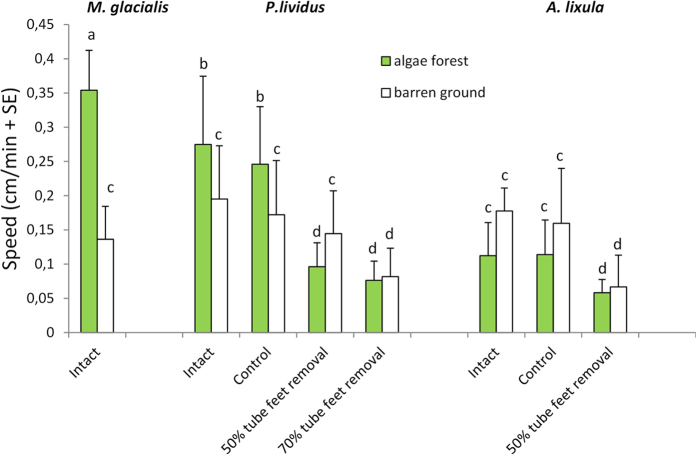
Attack speed (*M. glacialis*) and escape speed (*P. lividus* and *A. lixula*) measured in intact individuals (normal), control and treated individuals (with 50% and 70% tube feet removed) in forest (green bars) and barren (empty bars). A 3-way ANOVA was performed and significant effect of the term “Treatment X Habitat” was found (Table 1). SNK a posteriori tests were run to investigate the differences found (Table 1); similar letters (a, b, c, d) indicate groups of data not significantly different. In forest, *M. glacialis* was faster than *P. lividus* and *A. lixula* (normal, intact, control and treated). In barren, *M. glacialis* was faster than *P. lividus* and *A. lixula* when 70% and 50% tube feet, respectively, were removed (Table 1). We observed that *M. glacialis* is able to “float” above the forest thanks to its long arms, while urchin movement was affected by their spines tangling among the thalli of erect algae (Table 1).

The 3-way ANOVA analysis showed a significant effect of the term “Treatment X Habitat” (Table 1). SNK *a posteriori* tests were run to investigate the differences found (Table 1, Fig. 2). In the forest, *M. glacialis* was always faster than *P. lividus* and *A. lixula*. We observed that the seastar was able to “float” above the forest thanks to its long arms, while urchin movement was impeded by their spines tangling among the thalli of erect algae.

In the barrens habitat, the speed of sea urchins was negatively affected only when at least 70% and 50% of tube feet were removed from *P. lividus* and *A. lixula*, respectively (Table 1; Fig. 2).

**Figure 3:**
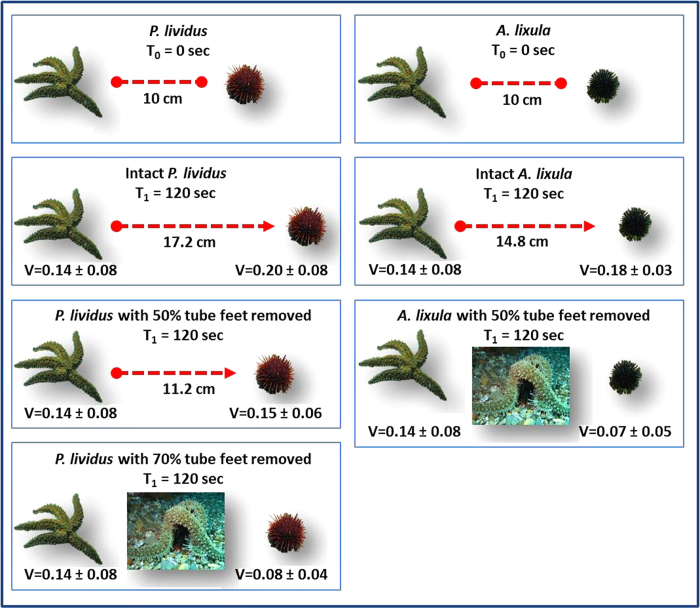
Simulation of the effect of tube feet removal on escape velocity and predation outcome on the barren habitat based on experimental data (Table 1, Fig. 2). V = Mean velocity (±s.e.). of attacking *M. glacialis* and escaping sea urchins (*A. lixula* and *P. lividus*). Removal of 70% and 50% of tube feet respectively in *P. lividus* and *A. lixula* allows the predator *M. glacialis* to reach and consume them. Simulated reaction distance of sea urchins is 10 cm (images in figure by F. Di Trapani).

The removal of tube feet slowed the movement speed of sea urchins, allowing *M. glacialis* – which otherwise moved more slowly than the urchins in barrens habitat – to overtake and capture them (Fig. 3).

## Discussion

The hypothesis that a synergistic facilitative interaction between the seastar *M. glacialis* and the fish *T. pavo* makes sea urchins vulnerable to seastar predation was corroborated by the results of this study. Sea urchin resulted the preferred food item of *M. glacialis* while sea urchin tube feet were frequently found in the *T. pavo* gut contents.

The experimental removal of sea urchin tube feet was quantitatively similar to the action of wrasses during the escape behaviour triggered by the seastar. Our results suggested that *T. pavo* facilitated *M. glacialis* in the barren habitat by removing tube feet and reducing escape speeds of sea urchins allowing higher rates of capture by *M. glacialis*. At the same time, *T. pavo* seems to have benefited from the escape response of sea urchins triggered by the seastar, since extended tube feet became accessible to the fish. However, in our study we lacked the data to demonstrate whether the absence of seastars affects the ability of *T. pavo* to capture sea urchin tube feet.

The establishment of a facilitative interaction between *T. pavo* and *M. glacialis* is particularly interesting since these species usually occupy non-overlapping habitats, and neither of them generally feed on adult of sea urchins^42^,^43^. The simultaneous occurrence of sea urchins, *M. glacialis* and *T. pavo* in the shallow rocky shore of Ustica, where high densities of the seastar occurred^14^, likely created the condition for the described interaction to happen.

Species of the *Thalassoma* genus display different feeding strategies. This fish can use stones as anvils to crash bivalve that are too large to swallow; it acts as facultative cleaner-fish; it follows “nuclear fish ” eating particles stirred up from the bottom^44^; and it follows divers to obtain food^53^. Importantly, wrasses are able to remember what, where and when food can be found^54^. Data from gut contents of *T. pavo* at Ustica clearly showed that sea urchin tube feet are an important component of its diet. Hence, *T. pavo* may have “learned” to follow the attacks of *M. glacialis* on sea urchins in order to easily feed on the sea urchin tube feet. This is of particular interest since *T. pavo* is a thermophilic species and its Mediterranean range has moved progressively northward, following increases in water temperature^55^. It is possible that interactions between *T. pavo* and seastars or other weak sea urchin predators will arise in other regions, with important consequences on sea urchin populations and benthic community structure.

The correlation between the decrease in sea urchin abundance, the increase in seastar population and the recovery in algae forest (Fig. 1) indicates that the high predation on sea urchins by *M. glacialis*, facilitated by *T. pavo*, is a key process for the recovery of the algae forest at Ustica island^14^,^40^.

The establishment of MPAs is a widespread tool for marine managers to fight the detrimental effect of multiple stressors, such as loss of species and climate change, on coastal systems worldwide^11^. Several studies indicate a positive effect of protection on targeted species within MPAs and, in some cases, the recovery of former ecosystems (e.g., kelp forest) through trophic cascades^33^,^35^,^36^. In other cases, however, the response of communities to protection seems to be idiosyncratic^10^. The variability in the effects of protection could depend on the time scale considered, as both direct and indirect effects of protection are slow, unstable and asynchronous^16^. Moreover, even if the goal of MPAs is the protection of the whole assemblages of organisms and their interactions, monitoring programs often focus only on targeted and conspicuous species, or limited assemblages (e.g., fish assemblages). The potential role of other species and the unique assemblages that may result due, in part, to their establishment, on the dynamics of protected ecosystems is thus largely overlooked.

Predator diversity may enhance the strength of trophic cascades by providing alternative consumers when environmental conditions change (i.e., Insurance Hypothesis)^56^,^57^. However, many systems are characterized by key predators and weak alternative consumers, whose effect on prey populations is mild^58^,^59^. In other cases, multi-predator assemblages may alter the strength of trophic control on preys by the so-called emergent multi-predator effect. Different predators can establish synergistic interactions, either positive (i.e., facilitation) or negative (i.e., interference), affecting predation rate on common prey^19^,^59^.

**Figure 4:**
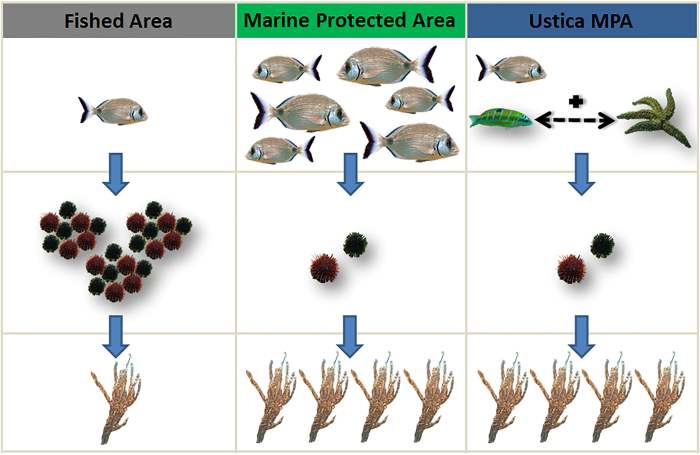
Conceptual model of the trophic cascades without the presence of MPAs, in their presence and in the Ustica case study. In areas where fishing is allowed, low densities of *Diplodus* causes the outbreak of sea urchin and consequently barren grounds are common. MPAs with their no-take policy are instead characterized by high densities of *Diplodus* that control urchins and allow algal canopy to return. The peculiar case of the Ustica MPA consists instead of low densities of *Diplodus* but, at the same time, extended algal coverage and low densities of sea urchins. In this case the seastar *M. glacialis* interacts with the ornate wrasse *T. pavo* and thus is able to heavily and successfully prey upon urchins species *P. lividus* and *A. lixula* and control their populations (images in figure by C. Bonaviri, F. Di Trapani and P. Gianguzza).

Here we presented a case where the paucity of traditional key predators is compensated by an unexpected interaction between two weak predators (a seastar and a fish), leading to the ecosystem restoration (Fig. 4). More broadly, our study represents an example where predator assemblage diversity strengthened the trophic control of keystone grazers, allowing the recovery of the algal forest habitat (i.e. the three-dimensional component).

Our results confirm the importance of long term studies for proper assessment of the direct and indirect effects of protection on ecosystems. This study is also a reminder of the importance of field observation for revealing arising interactions among novel species assortments in a changing community.

## Methods

### Study area

“Ustica Island” MPA is located on a small volcanic island 80 km off the northern coast of Sicily, covering 16,000 ha, of which 65 are devoted to a no-take area along the northwestern part of the island. Like other volcanic islands, Ustica is characterized by steep cliffs, which favor the abundance of the thermophilic wrasse *T. pavo* within its distribution range^48^. The density of *T. pavo* is high at Ustica with estimates ranging from 48.6 mean individuals/250 m^2^ (Galasso unpublished data) to 140.33 mean individuals/250 m^2^ 60. In the framework of scientific monitoring projects on the benthic megafaunal assemblages (2003-2010), an interaction between *T. pavo* and *M. glacialis* feeding on sea urchins was repeatedly observed. Ustica also has an unusually high abundance of the seastar *M. glacialis* with 1.2 mean individuals/250 m^2^ unlike other Mediterranean areas^14^ ranging from 0 to 0.1 mean individuals/250 m^2^ 14.

### Long-term monitoring of algae and sea urchins

Erect macroalgae cover (i.e., filamentous, foliose, corticated foliose, corticated macrophytes, leathery macrophytes and articulated calcareous) was visually estimated by either stereomicroscope analysis of slides taken by an analogue camera at high resolution, or computer analysis of images captured by a digital camera. A grid of 24 small quadrats was superimposed over each image and the abundance of each taxon assessed by giving a score from 0 to 4 to each subquadrat^61^. The total density of sea urchins (>1 cm test diameter) was estimated by visual counts within 1 m^2^ quadrats^39^. We analyzed 40 images for erect macroalgae cover and 40 replicates of each species of sea urchin per year.

### Analysis of *T. pavo* gut content

In order to evaluate the frequency of tube feet ingestion by fishes, we collected 40 *T. pavo* individuals between June and July 2008, and analyzed their gut contents in the laboratory. Total fish length was measured to the nearest millimeter and stomachs were removed and fixed in 5% buffered formalin for gut content analysis. For those stomachs containing food, we calculated the percentage frequency of those containing tube feet and then the number of tube feet were quantified under a binocular microscope^62^. Fish size ranged from 8 to 12.5 cm (10.5 ± 1.3 cm, mean ± s.d.). Only four individuals had an empty stomach.

### Prey of M. glacialis

*M. glacialis*, like many other seastar species, consumes its prey by extruding its stomach to envelop prey and digesting the soft tissues. We estimated *M. glacialis* prey frequencies in the field by lifting individuals, turning them upside-down and identifying the prey item to the lowest possible taxonomic level^63^. We conducted observations in 50 × 5 m random transects at ca. ≈5 m depth. Each individual showing feeding activity was measured using a Vernier caliper (maximum tip-to-tip diameter or total length, *T*_*L*_). We observed 241 *M. glacialis,* of which 118 were feeding upon 11 different prey items (Supplementary Table 1). Surveys were performed from June 2008 to June 2010. Data were grouped into 9 size classes: I (16–17 cm), II (17.1–20 cm), III (20.1–22 cm), IV (22.1–25 cm), V (25.1–27 cm), VI (27.1–29 cm), VII (29.1–32 cm), VIII (32.1–34 cm) and IX (>34 cm TL). To evaluate the effect of seastar size on its diet, we performed cluster analysis on a Bray Curtis similarity resemblance matrix of the arcsine-transformed data (Primer E Software v.6 64). Cluster analysis showed a clear division of seastars into two groups, larger and smaller than 27 cm *T*_*L*_(Supplementary Table 1), depending on their diet.

### Field observation of tube feet removal by *T. pavo*

The number of urchin tube feet removed by *T. pavo* during the attacks by *M. glacialis* was estimated in the field by video analysis of predation experiments. For these experiments we placed seastars in the field where sea urchins were naturally present and abundant. Seastar individuals had been previously starved for at least two weeks. We used two different methods to video-record the attacks. In one case, three video cameras (JVC EVERIO GZ-MG330 with 30 GB internal hard-drive, equipped with a modified waterproof case connected to long-life battery packs) were secured through tripods on the sea floor, for remote recording. Alternatively, cameras were directly operated by scuba divers. The number of urchin tube feet removed by *T. pavo* during each seastar attack on a sea urchin was subsequently evaluated by computer video analysis. The duration of the attacks ranged from a number of seconds to a few minutes, and terminated when the seastar reached the sea urchin or spontaneously stopped. Experiments were run in summer 2009.

### Effect of habitat on attack speed of *M. glacialis* and on escape speed of treated and untreated sea urchins

To test whether the removal of tube feet effectively compromised the sea urchin movement, we simulated the effect of the wrasse bite experimentally, by removing tube feet from individuals collected in the field. We then measured the attack speed of *M. glacialis* and the escape speed of both intact and impaired sea urchins (*P. lividus* and *A. lixula*) in the field. The tested habitats were barren ground, where the four species are abundant, and algal forest. In order to test the effect of the habitat on the speed of sea urchins and *M. glacialis* and the effect of tube feet removal on sea urchins speeds we manipulated the number of sea urchins tube feet in laboratory. Individuals of both *P. lividus* (n = 48) and *A. lixula* (n = 48) were placed in a glass tank in the laboratory. When the sea urchins were properly attached and adhered to the substratum, we removed the tube feet by rapidly pulling the urchins off the tanks. The detached tube feet remained on the wall of the tank and were easily counted. Using the equation of Santos & Flammang^65^ for estimating the total number of tube feet present in an urchin and by repeating the operation described above, we proceeded with the removal of 50% and 70% of tube feet for both *P. lividus* and *A. lixula*. Manipulated urchins were then used in the field to run the tests. To check for differences between treated and non-treated urchins due to handling or transportation stress, we also ran control tests on non-manipulated sea urchins. We performed a 3-way ANOVA analysis considering three factors^66^. Treatment, fixed with 8 levels: untreated *M. glacialis*, untreated *P. lividus*, untreated *A. lixula*, *P. lividus* with 50% of tube feet removed, *A. lixula* with 50% of tube feet removed, *P. lividus* with 70% of tube feet removed, control for *P. lividus* and control for *A. lixula*; Habitat, fixed with 2 levels: forest and barren; Site, with 2 levels, random and nested in Habitat. We ran 6 replicates for each experimental block. The sizes of the experimental animals were as follows: 4.28 ± 0.4 cm (mean ± s.d.) for *P. lividus*, 4.36 ± 5 cm (mean ± s.d.) for *A. lixula* (mean ± s.d.) and 32.2 ± 8 (mean ± s.d.) cm for *M. glacialis*. Experiments were run in summer 2009.

### Ethic statement

For sea urchin and seastar collection and manipulation, no permits were required according to national laws (Italian Legislative decree n. 116/1992). All experimental protocols were approved by the Department of Hearth and Marine Science (DiSTeM) of the University of Palermo. This research was conducted under a research permit from MPA local authority (0041110-26/05/2010 – Harbour Office of Palermo, MPA management body of the Ustica Island).

## Additional Information

**How to cite this article**: Galasso, N. M. *et al.* Fish-seastar facilitation leads to algal forest restoration on protected rocky reefs. *Sci. Rep.* **5**, 12409; doi: 10.1038/srep12409 (2015).

## Supplementary Material

Supplementary Information

## Figures and Tables

**Table 1:** Two-way ANOVA on attack speed (*M. glacialis*) and escape speed (*P. lividus* and *A. lixula*).

Source	DF	MS	F	P	F(versus)
Treatment (Tr)	7	0.1129	44.65	0.0000	SpxSi (Ha)
Habitat (Ha)	1	0.0278	19.63	0.0473	Si (Ha)
Site (Ha)	2	0.0014	0.38	0.6835	RES
TrxHa	7	0.0544	21.52	0.0000	SpxSi (Ha)
TrxSi (Ha)	14	0.0025	0.68	0.7897	RES
RES	160	0.0037			
TOT	191				

Cochran Test C = 0.1036; p > 0.05 no transformation. SNK on the term TrXHa. Within Habitat. Barren (MG = PL −50% = AL CTRL = PL CTRL = AL = PL > AL −50% = PL −70%). Forest (MG > PL CTRL = PL > AL −50% = PL −70% = PL −50% = AL = AL CTRL).

Factors: Treatment, with 8 levels: intact *M. glacialis (*MG), intact *P. lividus* (PL), intact *A. lixula* (AL), *P*. *lividus* with 50% of tube feet removed (PL −50%), A. *lixula* with 50% of tube feet removed (AL −50%), P. *lividus* with 70% of tube feet removed (PL −70%), control for *P.lividus* (PL CTRL) and control for *A. lixula (*AL CTRL); Habitat, with 2 levels: forest and barren and Site, with 2 levels.
