# Supplementary material for: Fish-seastar facilitation leads to algal forest restoration on protected rocky reefs
Source: Sci Rep. 2015 Jul 22;5:12409. doi: 10.1038/srep12409 (PMC4510527; doi:10.1038/srep12409)
Supplement: Supplementary Information [file srep12409-s1.doc]

Fish-seastar facilitation leads to algal forest restoration on protected rocky reefs

Nicola M. Galasso^1^, Chiara Bonaviri^2^*, Francesco Di Trapani^2^, Mariagrazia Picciotto^2^, Paola Gianguzza^2^, Davide Agnetta^2^, Fabio Badalamenti^1^

**ADDITIONAL INFORMATION**

|  | **F**% | |
| --- | --- | --- |
|  | ***T_L_* ≤ 27 cm (N=39)** | ***T_L_* > 27 cm (N=79)** |
| *Paracentrotus lividus* | 5.06 | 51.28 |
| *Arbacia lixula* | 2.53 | 28.21 |
| Arciidae | 49.37 | 15.38 |
| *Hexaplex trunculus* | 7.59 | 2.56 |
| Columbellidae | 11.39 | 0 |
| Trochidae | 8.86 | 0 |
| Gastropoda | 5.06 | 0 |
| *Cerithium* spp. | 5.06 | 0 |
| Buccinidae | 3.80 | 0 |
| Cypraeidae | 0 | 2.56 |
| *Patella* spp. | 1.27 | 0 |

**Supplementary Table 1
*M. glacialis* diet**. Frequency of occurrence (F%) of different prey items from 118 observations. Individuals with *T_L_* ≤27 cm preferred bivalves Arciidae, including *Arca noae* and *Barbatia barbata* and gastropods and individuals with *T_L_* >27 cm preferred the sea urchin *P. lividus* and *A. lixula* with 51.3% and 28.2% of frequency of occurrence, respectively.

**
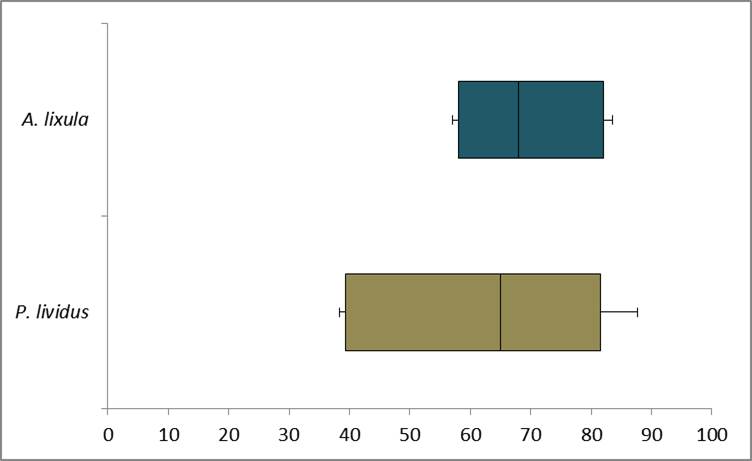
**

**Supplementary Fig.1
Percentage of tube feet removed from *A. lixula* (n=8) and *P. lividus* (n=9) by *T. pavo* during attacks by *M. glacialis.***Box: maximum, upper quartile, median, lower quartile and minimum.
